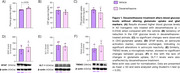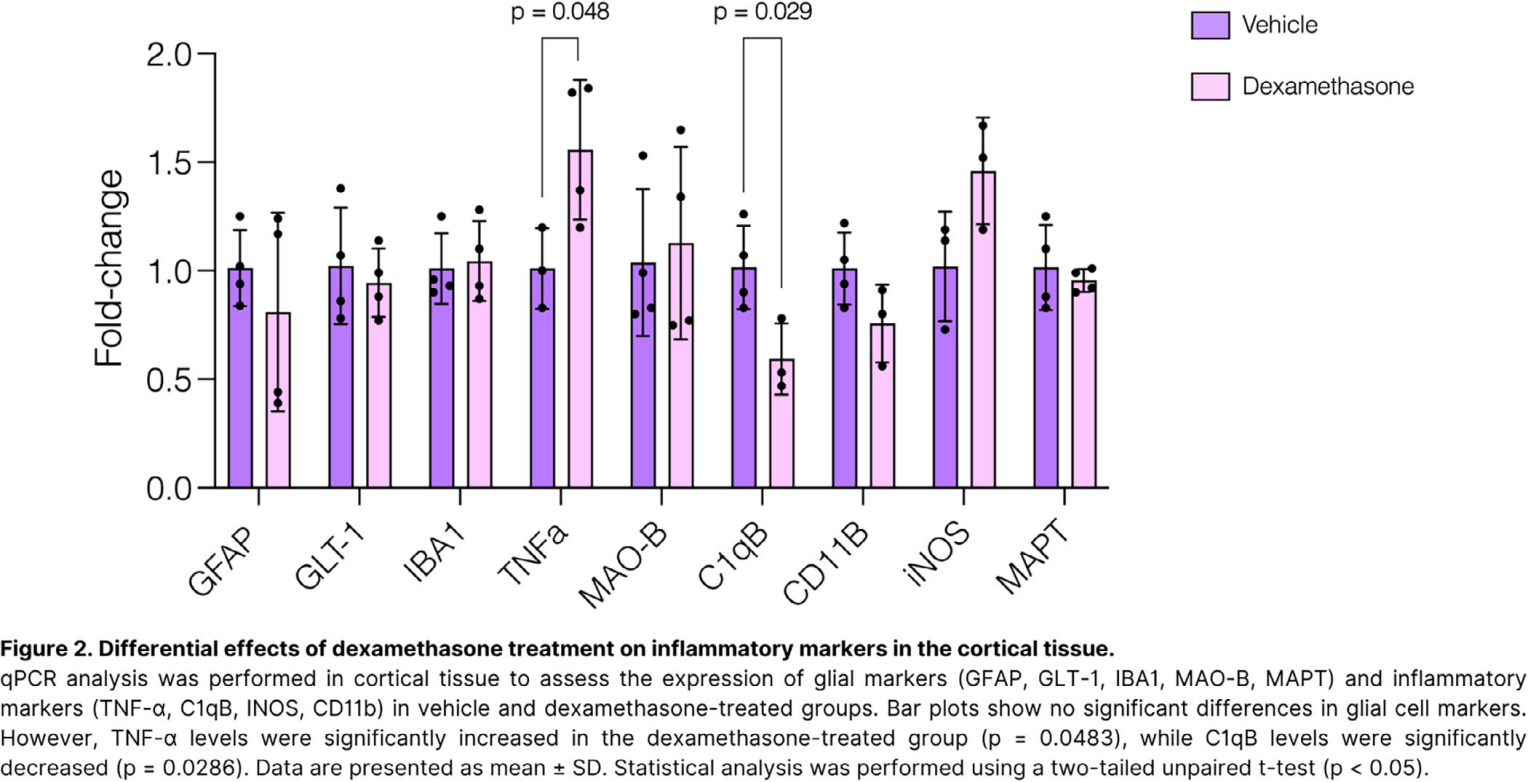# Impact of Glucocorticoids on Neuroinflammation in the TgF344‐AD Rat Model

**DOI:** 10.1002/alz70855_103076

**Published:** 2025-12-23

**Authors:** Roberta dos Santos de Oliveira, Christian Limberger, Gabriel Colissi Martins, Gabriel Lermen Hoffmeister, Mariana Radaelli Schmaedek, Ramon Bertoldi de Souza, Gabriela Mantovani Baldasso, Rodrigo Sebben Paes, Eduardo R. Zimmer

**Affiliations:** ^1^ Universidade Federal do Rio Grande do Sul, Porto Alegre, Rio Grande do Sul, Brazil; ^2^ Universidade Federal do Rio Grande do Sul, Porto Alegre, RS, Brazil; ^3^ McGill University, Montreal, QC, Canada; ^4^ Brain Institute of Rio Grande do Sul ‐ Pontifícia Universidade Católica do Rio Grande do Sul, Porto Alegre, Rio Grande do Sul, Brazil

## Abstract

**Background:**

Glial fibrillary acidic protein (GFAP) levels, a key marker of astrocyte reactivity, increase in response to Alzheimer's disease (AD) pathology, but its role in AD‐related neuroinflammation remains unclear. Dexamethasone, a glucocorticoid, modulates inflammation by binding to glucocorticoid receptors, suppressing pro‐inflammatory cytokines and immune cell activation. This study investigates the anti‐inflammatory effects of glucocorticoids on astroglial and microglial markers in the TgF344‐AD rat model.

**Method:**

Male TgF344‐AD rats (*n* = 8), in an early amyloid plaque stage (7‐8 months), received 0.25 mg/kg of dexamethasone (i.p.) for 14 days. After treatment, blood, cerebrospinal fluid (CSF), and brain tissue were collected. We performed [3H]‐glutamate uptake assay on acute cortical tissue slices and analyzed the cortical immunocontent and expression of inflammatory, astroglial, and microglial activation markers. Additionally, plasma and CSF glucose levels were measured with a colorimetric assay. Data were analyzed using Student's t‐test (*p* <0.05).

**Result:**

We observed higher plasma glucose levels in dexamethasone‐treated animals, confirming the systemic treatment efficacy (*p* = 0.0104) (Figure 1A). Additionally, there was a tendency of reduction in CSF glucose levels (Figure 1B). No significant changes in glutamate uptake and glial activation markers were observed (Figure 1‐2). However, the cortical expression of C1qB was reduced in dexamethasone‐treated animals (*p* = 0.02) and, contradictory, increased in TNF‐α was observed (*p* = 0.04) (Figure 2).

**Conclusion:**

Our initial results indicate that, despite no changes in glial marker levels, the treatment reduced C1qB levels while paradoxically increasing TNF‐α in the TgF344‐AD model. Since both TNF‐α and C1qB are predominantly secreted by microglia, further studies are warranted to investigate whether dexamethasone induces a distinct microglial phenotype. Additional experiments are necessary to determine if these changes signify a shift in immune balance and whether they have protective or detrimental effects.